# A bilateral sinus cyst treated via a bilateral frontonasal bone flap in a standing horse

**DOI:** 10.4102/jsava.v90i0.1729

**Published:** 2019-03-28

**Authors:** Mickaël P. Robert, Gideon P. Stemmet, Yolandi Smit

**Affiliations:** 1 Department of Companion Animal Clinical Studies, Faculty of Veterinary Science, University of Pretoria, Onderstepoort, South Africa

## Abstract

Bilateral paranasal sinus diseases are rarely reported in horses. Treatment using a bilateral frontonasal bone flap on a standing, regular-sized adult horse has not been described previously. A 13-year-old Thoroughbred gelding was evaluated for bilateral mucopurulent nasal discharge. Radiographic, endoscopic and computed tomographic examinations revealed bilateral sinus pathological changes consistent with an ethmoid haematoma involving the maxillary and frontal sinuses. A bilateral frontonasal bone flap was created under standing sedation and local anaesthesia. A tracheotomy was performed initially to ensure a patent airway during the procedure. Additional analgesia had to be given to transect the dorsal part of the nasal septum while elevating the flap. The sinus masses were removed and communications with the nasal cavities created uneventfully. Small sequestra were removed transendoscopically from the left caudal maxillary sinus 4 weeks after the initial procedure. The horse made a complete recovery with an excellent cosmetic outcome. Histopathology revealed the mass to be a sinus cyst. We concluded that a bilateral sinus bone flap can be used in adult regular-sized horses to access the left and right paranasal sinuses simultaneously. Regional nerve blocks should be performed in order to increase analgesia. A temporary tracheotomy prevents any airway obstruction during the procedure. The post-operative cosmesis is excellent.

**Keywords:**

frontonasal bone flap; sinus cyst; computed tomography; standing surgery; horse.

## Introduction

Bilateral paranasal sinus diseases are rarely reported in horses. In previous studies reporting on large numbers of horses with sinonasal diseases, only 3.0% – 4.5% of cases had bilateral sinus involvement (Dixon et al. 2011; Tremaine & Dixon 2010). These cases may require bilateral sinus surgery in order to treat the primary affection. Even if the trend in treating sinonasal disorders in horses is to be as conservative and as minimally invasive as possible (Barakzai & Dixon 2014; Dixon et al. 2012b; Easley & Freeman 2013a), sometimes osteoclastic flaps remain essential to get sufficient access to the lesion. Performing these flaps on the standing horse decreases the costs for the client, minimises intraoperative bleeding, improves visualisation and alleviates the risks of general anaesthesia (Barakzai & Dixon 2014; Easley & Freeman 2013a). A first option to treat bilateral sinus conditions is to approach each side with a bone flap 3 weeks apart (Easley & Freeman 2013a). Alternatively, treatment using a large caudally based bilateral frontonasal bone flap has recently been described on a standing miniature horse with a bilateral mucocoele (Easley & Freeman 2013b). However, to our knowledge, such a procedure has not been reported on a fully grown regular-sized horse.

## Ethical considerations

The owner of the horse gave written consent for the surgery to be performed. The Research Ethics Committee of the university approved this case report (project REC054-18).

## Case presentation

A 13-year-old Thoroughbred gelding was presented to the Onderstepoort Veterinary Academic Hospital with a 1-month history of unilateral, mucopurulent nasal discharge that progressed to a bilateral nasal discharge 1 week prior to presentation. Upon clinical examination, all vital parameters were within normal limits. A moderate bilateral facial swelling was noted over the caudal maxillary sinuses as well as a bilateral purulent nasal discharge. Shallow infundibular caries were observed on teeth 109, 110, 209 and 210.

**FIGURE 1:**
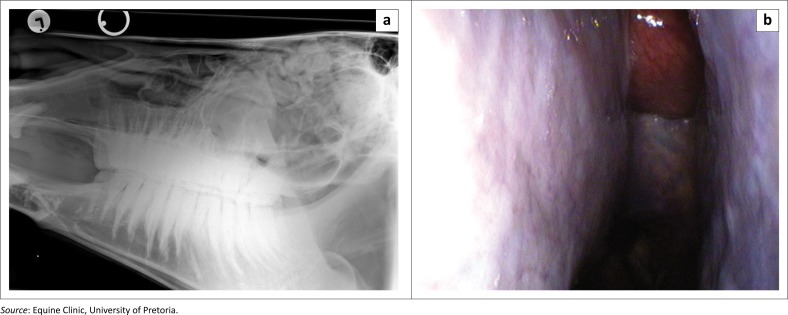
Initial ancillary diagnostic examinations of a 13-year-old Thoroughbred gelding presented with a bilateral nasal discharge. (a) Lateral head radiographs. Note the well-circumscribed soft tissue masses situated in the dorsal conchal sinuses and the fluid lines present within the caudal maxillary sinuses. Additional soft tissue opacities are visible in the ethmoidal region. (b) Endoscopic view of the left middle meatus. Note the red round mass extending from the left ethmoid region rostrally into the nasal cavity.

Radiographic examination showed a round, well-circumscribed soft tissue opacity situated in the left ethmoid region extending rostrally into the nasal passages as well as towards the right side. Fluid was present within both caudal maxillary sinuses. The left ventral conchal sinus appeared enlarged with increased radio-opacity. Additional masses were visible in the dorsal conchal sinuses (Figure 1a).

Upper respiratory endoscopy revealed a reddish globulous mass, compatible with an ethmoid hematoma, extending from the left ethmoid region rostrally into the nasal cavity, occluding the left sinus drainage angle (sinonasal drainage aperture) (Figure 1b). Mucopurulent discharge was present in both nasal passages as well as at the right sinus drainage angle (Barakzai & Dixon 2014).

**FIGURE 2:**
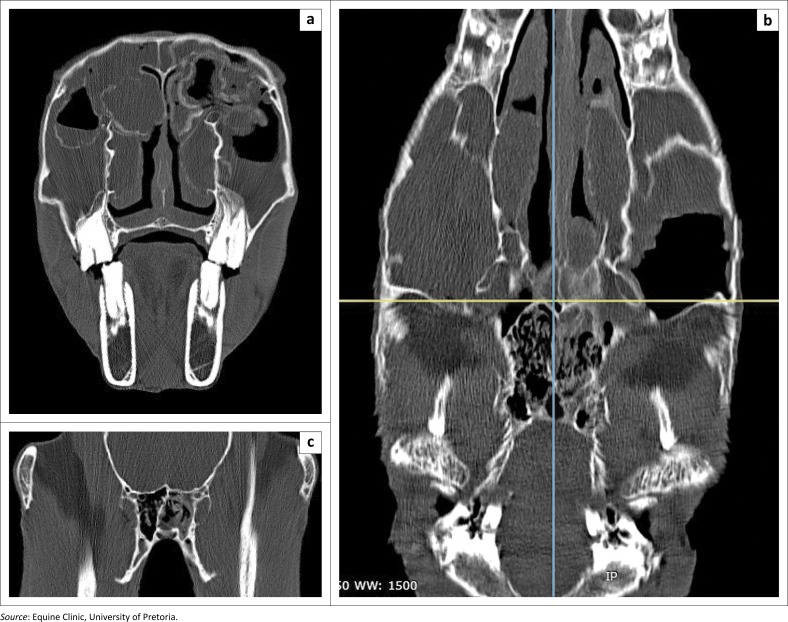
Computed tomography study of the affected horse’s head. (a) Transverse image at the level of teeth 110 and 210. (b) Frontal image, parallel to the dorsal surface of the frontal bones, at the level of the ventral orbit. (c) Transverse close-up view of the sphenoidal sinus. Note the extensive bilateral fluid accumulation in the rostral and caudal maxillary sinuses, ventral and dorsal conchal sinuses, left sphenopalatine and left ethmoidal sinuses, with some fluid accumulations contained in partially mineralised walls.

Computed tomography (CT) performed under standing sedation (Siemens Somatom Emotion Duo, Siemens, Erlangen, Germany) revealed extensive bilateral fluid accumulation in the rostral and caudal maxillary sinuses, ventral and dorsal conchal sinuses, sphenopalatine sinuses as well as the left ethmoidal sinus, sometimes contained by mineralised walls. Discrete masses were obvious in the left dorsal conchal sinus. These findings were consistent with widespread bilateral ethmoid haematomata (Figure 2).

**FIGURE 3:**
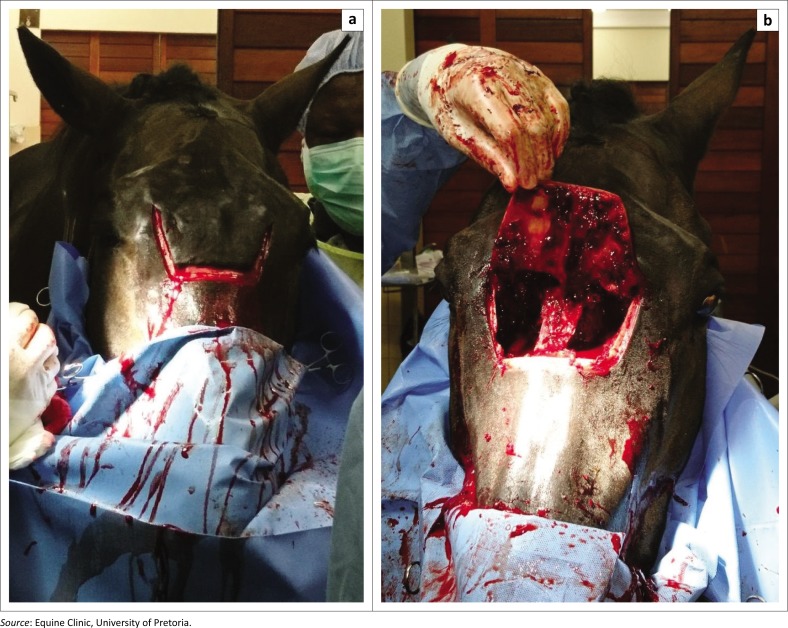
Intraoperative photographs during creation of a large bilateral frontonasal bone flap on a standing horse. (a) Skin incision performed according to the described boundaries. (b) Image taken after extirpation of the abnormal sinonasal tissues and before packing. Note the excellent surgical exposure gained through this flap.

Three days after presentation, the horse underwent surgery under standing sedation using detomidine (Domosedan, Zoetis, Sandton, South Africa; 10 *µ*g/kg, intravenously [IV] as a bolus followed by an infusion at 0.5 *µ*g/kg/minute initially and then titrated to effect) and morphine (morphine 10 mg injection, Pharma-Q, Johannesburg, South Africa; 0.1 mg/kg, IV). Single dosages of flunixin meglumine (Finadyne, MSD, Kempton Park, South Africa; 1.1 mg/kg, IV) and intramuscular procaine penicillin (Depocillin, MSD, Kempton Park, South Africa; 22 000 IU/kg) were administered. In anticipation of surgical haemorrhage and associated packing, a temporary tracheotomy was performed preoperatively to ensure a patent airway (Barakzai & Dixon 2014; Nickels 2012). A large bilateral frontonasal sinus bone flap was performed, according to Easley and Freeman (2013b) using an oscillating saw. Local anaesthesia was achieved through subcutaneous infiltration of 2% lidocaine (Lignocaine Fresenius, Fresenius Kabi, Midrand, South Africa) on the incision sites. Briefly the lateral limit was a line starting 2 cm axial to the medial canthus of the eye, halfway between the medial canthus and the supraorbital foramen, extending to approximately 2/3 the distance from the medial canthus to the infraorbital foramen, staying parallel and dorsal to a line between the medial canthus and the nasoincisive notch (Freeman et al. 1990; Nickels 2012). The rostral limit connected the rostral aspect of both lateral incisions (Figure 3a). Topical lavage and injection of lidocaine 2% was performed to provide additional analgesia during the dorsal nasal septum osteotomy, where a large flat osteotome was used to reflect the bone flap caudally. This approach allowed excellent exposure of both left and right sinus cavities (Figure 3b).

Masses consistent with ethmoid haematomata were removed from both frontal and caudal maxillary sinuses using traction with sponge forceps and digital separation, and specimens thereof were submitted for histopathology. Maxillary septal bullae were digitally perforated and these communications enlarged with arthroscopic rongeurs (Dixon et al. 2012a, 2015). Communication between the medial wall of the dorsal conchal sinus and middle meatus was created to ensure adequate drainage and allow future endoscopic evaluation of the sinuses (Nickels 2012). Substantial bleeding was observed. In order to provide haemostasis, the sinuses were lavaged using cold sterile saline and packed with sterile stockinette exiting and fixed to both nostrils (Nickels 2012). The flap was repositioned digitally and the incision closed in two layers and protected by an adhesive bandage for 7 days. Antibiotic treatment (Purbac, Pharmacare Limited, Port Elizabeth, South Africa; 25 mg/kg, PO) was pursued for 10 days. Anti-inflammatory medicines (Finadyne, MSD, Kempton Park, South Africa; 1.1 mg/kg, IV) were given at tapering doses for 6 days (every 12 hours for 3 days and then once a day for 3 days).

The sinus packings were removed 48 h post-operatively through the nostrils with the horse sedated. Because a piece of stockinette was left behind, the right caudal maxillary sinus had to be trephined to extract the latter using arthroscopic rongeurs and sinoscopic guidance (Nickels 2012). Skin staples were removed 11 days after surgery when the horse was discharged from the hospital.

Histopathological examination revealed the mass to be a sinus cyst with its wall being lined on both sides by a layer of pseudostratified respiratory epithelium. The connective tissue subjacent to the epithelium displayed various degrees of oedema and haemorrhage, both acute and chronic, granulation tissue formation and chronic inflammation. All cyst walls contained bony plates centrally that were made up of bony spicules that showed varying degrees of remodelling.

**FIGURE 4:**
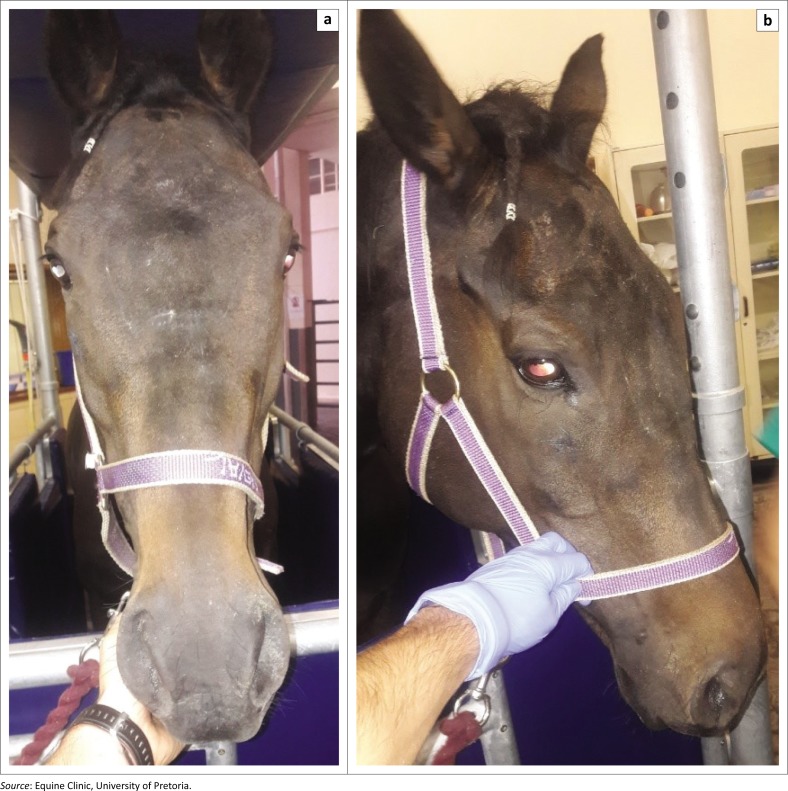
Photographs of the horse’s face 4 weeks after a large bilateral frontonasal bone flap was performed (a, b). Note the excellent cosmetic result.

Four weeks after surgery the horse was re-evaluated because of a mild left unilateral mucopurulent nasal discharge. The cosmesis of the surgical site was deemed excellent (Figure 4). The tracheotomy site was completely healed. Endoscopic examination revealed the presence of small sequestra in the left caudal maxillary sinus that were extracted using an endoscopic biopsy forceps.

Nine months after initial surgery, no recurrence of the nasal discharge was observed. Satisfaction of the client was excellent.

## Discussion

We described the successful surgical management of a bilateral sinus cyst using a large bilateral frontonasal bone flap on a standing adult Thoroughbred gelding.

The frontonasal bone flap was previously reported as being the ideal approach to the paranasal sinuses because it allows access to the conchofrontal sinus, the caudal maxillary sinus and the ventral conchal and rostral maxillary sinuses after perforation of the maxillary septal bulla (Easley & Freeman 2013a). With the bilateral frontonasal flap used in this case, excellent bilateral exposure was obtained to all these structures.

The patient experienced discomfort when we sectioned the dorsal nasal septum in order to lift the bone flap caudally, and we had to inject local anaesthetic onto the sinus mucosa and into the dorsal septum. Previous authors also injected local anaesthetic into the sinus before making bone flaps (Easley & Freeman 2013b; Schumacher et al. 2000). However because of the large size of the sinus cavities in adult regular-sized horses, it is likely that a regional block such as the maxillary nerve block, potentially combined with an ethmoidal nerve block, both performed bilaterally, would provide better analgesia of the nasal septum than local instillation and seems indicated for future cases (Caruso, Schumacher & Henry 2016; Easley & Freeman 2013a).

Although it was stated that because of the minimal blood loss occurring in standing sinus flap procedures, sinus packing may not be required (Easley & Freeman 2013a), we considered this step essential at completion of our procedure because of the substantial bleeding. Unfortunately, this packing tore during removal through the nostril, requiring an additional procedure to remove it.

Sequestration is a well-known complication of sinus surgeries. It can occur on the margins of the flap or into the sinuses. We did not experience any healing complication, compared to other cases (Easley & Freeman 2013b), perhaps because we completely sutured the periosteum and because our horse received systemic antibiotics (Dixon et al. 2012b). The duration of post-operative treatments and hospitalisation were similar to those reported by Fenner et al. (2019).

No post-operative sinus lavage was performed in our case. This could have prevented dehiscence from occurring because early aggressive lavage could lead to fluid leakage into the peri-incisional tissues (Barakzai & Dixon 2014). On the other hand, post-operative lavage might have reduced the chance of sequestrum formation through flushing of debris from the sinuses. It is believed that these sequestra came from the maxillary septal bulla fenestration or the sinonasal fistulation (Dixon et al. 2012a). Visualisation of these small pieces of bone was probably impaired because of the intraoperative bleeding but was then facilitated by the created sinonasal communication.

Even if radiographs and endoscopy were useful in confirming the bilateral nature of the affection, CT allowed a thorough three-dimensional evaluation of all the sinus cavities and permitted elaboration of the surgical plan, as previously reported (Barakzai & Dixon 2014; Manso-Díaz et al. 2015). Furthermore the ability to perform this examination on a standing horse also minimises the cost and anaesthetic risk.

Our initial diagnosis based on endoscopic and surgical findings was an ethmoid haematoma. However, histopathology revealed the mass to be a cyst. Sinus cysts generally carry an excellent prognosis, with only a single treatment usually required (Dixon et al. 2012b; Fenner et al. 2019). Some authors have previously suggested a common origin between sinus cysts and ethmoid haematomas (Lane, Longstaffe & Gibbs 1987; Nickels 2012), but the former has a much lower recurrence rate, about 19% (Fenner et al. 2019), compared to up to 43% for the latter (Nickels 2012). The CT appearance of this cyst, with fluid lines and focal mineralisation, is consistent with previous descriptions (Fenner et al. 2019; Manso-Díaz et al. 2015).

The aesthetic result of this large bilateral frontonasal bone flap was considered excellent and similar to what has been reported for unilateral maxillary and frontonasal bone flaps (Dixon et al. 2012b). Our case did not show any relapse of the disease.

In conclusion, it appears that a large bilateral sinus bone flap can be used successfully to access left and right paranasal sinuses simultaneously without jeopardising the horse’s cosmesis. A temporary tracheotomy should be considered if substantial intraoperative bleeding is expected. Regional nerve blocks should be considered to ensure adequate analgesia.
